# Mechanical Circulatory Support in Delayed Surgery of Post-Infarction Ventricular Septal Rupture in Patients in Cardiogenic Shock—A Review

**DOI:** 10.3390/jcm11164728

**Published:** 2022-08-12

**Authors:** Karolina Żbikowska, Krzysztof Wróbel

**Affiliations:** 1 Department of Cardiac Surgery, Medical University of Warsaw, 02-097 Warsaw, Poland; 2 Department of Cardiac Surgery, Medicover Hospital, 02-972 Warsaw, Poland; 3 Cardiac Surgery Clinic, Lazarski University, 02-662 Warsaw, Poland

**Keywords:** ventricular septal rupture, ventricular septal defect, delayed surgery, mechanical circulatory device, mechanical circulatory support, myocardial infarction

## Abstract

Post-infarction ventricular septal rupture (VSR) is a serious complication of myocardial infarction, which, in its natural course or treated medically, is related to high mortality rate. Surgical intervention remains the treatment of choice. Recent studies have shown that delayed surgery is related to better outcomes in comparison with urgent surgery; however, in many studies the impact of the patients’ initial hemodynamic status on the treatment outcomes often remains unclear. In this review, we analyze the outcomes of delayed surgical treatment of patients in cardiogenic shock in the course of post-infarction ventricular septal defect stabilized with preoperative use of mechanical circulatory support. We evaluate the importance of various types of mechanical circulatory devices (MCD), such as extracorporeal membrane oxygenation, Tandem Heart, Impella, and intra-aortic baloon pump (IABP) in preoperative stabilization of patients, and the most suitable time for surgery, and we also present the features of ideal MCD for patients with VSR. A search of Pubmed to identify studies concerning the use of MCD in patients in cardiogenic shock in the course of VSR qualified for delayed surgery was conducted in January 2022. A total of 16 articles with three or more patients described were analyzed in this study. The preoperative use of MCD in patients in cardiogenic shock and delayed surgery as a main part of treatment seems to be a promising direction, however, it requires further research.

## 1. Introduction

Ventricular septal rupture is a serious mechanical complication of myocardial infarction (MI) with an unfavorable prognosis. Observational studies have shown that delayed surgical treatment (especially longer than 7 days) is associated with better outcomes than urgent surgery [1]. However, it is not clear whether better survival is associated with better preoperative status, allowing patients to survive prolonged waiting time for surgery of more than 7 days, or postponing surgery itself. Many patients in cardiogenic shock in the course of VSR are unable to survive until delayed surgical intervention. Mechanical circulatory support may enable hemodynamic stabilization of the patient in shock and postpone the procedure, although this therapy can be associated with serious complications. The optimal duration of safe circulatory support in these cases as well as the type of MCS device used remain unknown. We analyze the recent studies of preoperative use of mechanical circulatory support, especially venoarterial extracorporeal membrane oxygenation, Impella, and Tandem Heart as a bridge to a delayed cardiac surgery.

### 1.1. Epidemiology

VSR is a serious, life-threatening complication typical of transmural MI, the incidence of which decreased due to early percutaneous revascularization strategies from 2% to about 0.25% of cases [2,3,4,5]. Mortality in the natural course of the disease as well as in a pharmacological treatment is similar and amounts approximately to 87–96% [6,7]. The presence of a cardiogenic shock and hemodynamical instability substantially worsen the prognosis [8].

### 1.2. Pathophysiology

VSR develops in the course of MI with an occlusion of the left anterior descending artery supplying the anterior two-thirds of the interventricular septum or the right coronary artery (less frequently the circumflex artery), providing blood to one-third of the posterior part. Acute VSD is usually related to sudden, severe ischemia in the course of total occlusion of the artery, which causes advanced, extensive necrosis [9]. It is emphasized that the time of VSD formation in the reperfusion era was reduced to 1 day from 3–5 days in the pre-thrombolytic period [9]. Inferior infarct usually causes the defect with complex structure [10]. It is formed as a result of dissection, commonly of the posterior and inferior parts of the interventricular septum, caused by the formation of a hematoma in the infarcted tissue after acute coronary syndrome [3,10]. Anterior infarcts are often a simple defect between the chambers at the same height, most often involving the apical segments [11]. However, in clinical practice, complex VSR at the medial anterior part of the interventricular septum may be diagnosed, which occurs as a result of occlusion of the left descending artery leading to extended anterior wall infarction. VSD can also appear later in the post-infarction period as a consequence of a thin muscle tissue rupture inside a septal aneurysm [12,13]. The ventricular septal defect can increase in size within several weeks due to changes in the tissues affected by the infarction [6]. The grade of an interventricular shunt depends on the dimension of the defect and the ratio of pulmonary to systemic vascular resistance as well as the post MI right and left ventricular function [3,11]. Reducing cardiac output increases systemic resistance and thus afterload, which increases ventricular overload and can lead to an enlargement of the shunt [3].

### 1.3. Surgical Treatment

Surgical intervention remains the treatment of choice, with a mortality rate ranging from 20 to 88%, depending on initial hemodynamic status [11,14,15]. There is no agreement concerning optimal time of cardiac surgery [15]. The 2013 American guidelines recommend an urgent operation [16]. Observational study has shown that delayed surgery is associated with significantly better results [7,11,17]. Arnaoutakis et al. analyzed the results of treatment of over 2800 patients and found that surgery performed within 7 days after myocardial infarction was associated with 54.1% mortality, whereas surgery performed after 7 days was associated with the mortality rate at 18.4% within the first 30 days [1]. However, delay of the operation may lead to further enlargement of the interventricular shunt or, especially in the case of hemodynamical instability, to preoperative death [15]. Mechanical circulatory support as a bridge to cardiac surgery may be an encouraging solution [17]; nevertheless, recent European guidelines emphasize that the use of mechanical circulatory devices in patients with heart failure or cardiogenic shock, due to possible complications, requires further research [18]. There are two main techniques for the surgical treatment of VSR: Daggett’s method, consisting of direct reconstruction of the septum, most often with the use of Dacron patches after previous infartectomy [19], and David’s procedure, associated with exclusion of tissues with complications from myocardial infarction by sewing a patch from the left ventricle [20].

### 1.4. The Role of Mechanical Circulatory Support

The type of impact on the cardiovascular system depends on the device used.

- An intra-aortic balloon pump is an easily accessible percutaneous implantable device that reduces the afterload (LV unloading only) and improves flow in coronary vessels. IABP could potentially help to unburden the left ventricle and reduce the left–right shunt, although with a poor effect, especially in unstable patients [21,22]. However, it is used in combination with other types of mechanical circulatory supports.
- LV-based Impella (LV to Ao; LV support and unloading) is a hemodynamically effective microaxial device that pumps blood from the left ventricle to the ascending aorta, generating flow over 5 L/min, which leads to significant direct unloading of the LV and increased cardiac output [23]. Reducing left–right shunt Impella decreases the right ventricle overload and pulmonary congestion, simultaneously posing a risk of shunt inversion and hypoxia of the central nervous system and myocardium; therefore, intensive monitoring is recommended [24]. The presence of VSR is considered a contraindication to implantation of this type of support due to the risk of aspiration of necrotic tissues, thus, some authors suggest using Impella with posterior VSR to reduce the possibility of embolization [24].
- Venoarterial ECMO (RA to femoral artery (FemA)—biventricular support with RV unloading)—supports systemic circulation (biventricular support), increases the level of arterial blood oxygen saturation, and ensures proper tissue oxygenation, which is essential in the setting of large left to right shunts, especially in a non-opening aortic valve [22,25]. This MCS unloads the right ventricle; however, at the same time, ECMO raises the left ventricular end-diastolic pressure and the total blood flow, which may contribute to overload LV and enlargement of the rupture [25,26]. ECMO with LV unloading is the simultaneous use of IABP or LV-located Impella with ECMO (i.e., Ecpella) [23,27]. Ecpella unloads LV, prevents the VSD enlargement, reduces afterload, and drains the right atrium, supporting the right ventricle. An interesting option is the applicability of left atrial venoarterial membrane oxygenation (LAVA ECMO biatrial) with transeptal located cannula, which at the same time drains the left and right atria [23]. Another effective method for indirect unloading of the left ventricle is the use of the pulmonary artery draining cannula.
- Tandem Heart (LA to FemA; LV support and unloading) is a percutaneous system with a continuous-flow centrifugal pump that generates a flow of about 5 L/min, which indirectly decompresses the left ventricle [28]. Placing the inflow cannula through the femoral vein in the left atrium requires a transseptal puncture, which may be a certain limitation in the use of this system [28]. Tandem Heart reduces LV end-diastolic pressure, although, similar to ECMO, it increases afterload due to return of blood through the outflow cannula to the femoral artery [28]. In the setting of VSR, there is a risk of shunt inversion due to intensive LV unloading as well as affecting the opening of the aortic valve [24].
- Left ventricular assist device (LVAD) (LV to aorta) is also a support in some centers for patients with post-infarction VSD. However, LVAD is used less frequently for short-term pre-operative stabilization of the patient. Post-infarction fragility of tissues may impede adequate implantation of the support and aspiration of necrotic tissues, which in some cases may lead to improper function of the pump [24]. With the more favorable location of the post-infarction area and the possibility of using LVAD, there is a risk of reversing the leak, which can be solved by using BiVAD [24].

The most common complications associated with the use of MCDs include lower limb ischemia, bleeding, and hemolysis [29]. The relatively safe type of device among the listed ones is the IABP [30]. Low cardiac output may be a significant complication of MCD in patients with VSD, especially in the case of insufficient left ventricular unloading.

### 1.5. An Ideal Mechanical Circulatory Device for a Patient with Post-Infarcted VSR

The analysis of individual MCDs leads to the creation of a hypothetical set of properties of the ideal device to support the circulation of unstable patients with VSR. The ideal appliance should be easily accessible, safe, for implantation, manageable, and cost effective. From a hemodynamic point of view, a balanced unloading of both the left and right ventricles is important in order to obtain the possibility of recovery of the infarcted tissues without the risk of enlargement of the rupture and shunt inversion, which could lead to desaturation and generalized hypoxia. Reducing the end-diastolic pressure would diminish the wall stress of LV. Afterload reduction would prevent permanent aortic valve closure and the formation of a thrombosis in the aortic root. The available data may indicate that the strategy of combining ECMO and IABP as well as ECPELLA may be therapeutically beneficial; however, this issue requires further research.

## 2. Materials and Methods

The data presented in the review come from studies found via PubMed in January 2022. There were no exclusions regarding the publication year of the study. Only free and paid studies in the English language were considered. The search terms were: Ventricular Septal Rupture OR Ventricular Septal Defect AND Extracorporeal Membrane Oxygenation OR Mechanical Circulatory Support OR Tandem Heart OR Impella. A total of 373 records were found. Preliminary analysis of abstracts allowed us to exclude duplicates, texts concerning VSR unrelated to MI, articles unrelated to ECMO, Impella, or Tandem Heart mechanical circulatory supports, as well as case reports describing fewer than three patients (if it was possible on the stage of analysis of the abstract). During full-text analysis, the publications with incomplete data about the preoperative use of ECMO and studies fulfilling previously noted exclusion criteria were rejected. Due to the narrative nature of the text and the diverse type of analyzed studies, it was not possible to strictly follow the PRISMA guidelines; however, we tried to implement them to the fullest extent possible.

Assuming that the patients in cardiogenic shock are patients with the hemodynamic profile described by the class 1 of the INTERMACS scale, it should be emphasized that, in the analyzed studies, the preoperative adoption of MCD was used also in patients who did not strictly meet these criteria. However, the authors often highlighted that patients qualified for preoperative MCD had an unfavorable initial hemodynamic profile. Due to limited data, a thorough analysis of the initial hemodynamic status of patients is not always possible.

The term “delayed surgery” has not been clearly defined in studies of the treatment of mechanical complications of myocardial infarction. It is used in relation to the postponement of the surgical treatment of the aforementioned complications, especially post-infarction VSD, which, according to recent scientific reports, may be associated with significantly more favorable treatment outcomes. The appropriate timing for the operative repair of the VSR is the subject of an ongoing discussion initiated by Arnaoutakis in 2012 [1]. The postponement of surgery in patients in cardiogenic shock is often associated with the need for the use of mechanical circulatory support. The appointment of an operation date is an attempt to reach a compromise between achieving the time necessary to obtain hemodynamic stability and the attempt to avoid serious complications of mechanical circulatory support.

## 3. Results

### 3.1. Research Process

**Figure 1:**
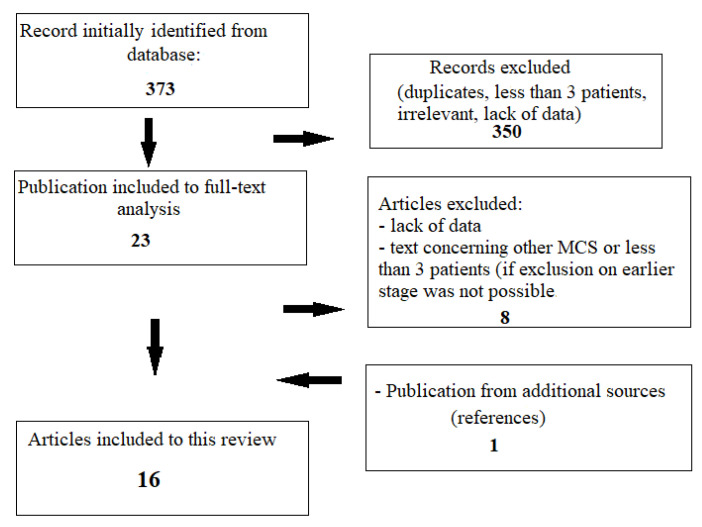
Research process.

Observational studies, reviews with elements of meta-analysis, and case series with more than three patients with ECMO, Impella, or Tandem Heart as a bridge to delayed surgery of VSR were used in this study, for a total of 15 publications. Additionally, at this stage, the references of selected articles were screened in order to find significant studies that may have been omitted during the search process. Finally, 16 articles were included in this work (Figure 1) and are summarized in Table 1.

### 3.2. Mechanical Circulatory Support in Delayed Surgery of Post-Infarction VSR—Results

Morimura et al. reviewed eight patients in cardiogenic shock in the course of VSR [17]. All of them had received percutaneous coronary intervention before the diagnosis. In order to obtain hemodynamic and metabolic stabilization, preoperative IABP was used in each patient; five of them received V-A ECMO. The degree of stabilization and myocardial recovery was assessed by analysis laboratory data. Median time from MI to surgery was 7.1 days; from VSR diagnosis to operation 1.9 days; and durations of mechanical circulatory support were 43.2 h in the setting of IABP and 36.9 h in the case of V-A ECMO, respectively. The perioperative mortality was 12.5%, whereas the 2-year mortality was about 37.5%. Out of five patients who received ECMO preoperatively, one patient died in the perioperative period, and one patient experienced a minor bleeding complication.

Similar mortality was reported by Malik J. et al. [30]. Out of 27 patients with post-infarction VSR in cardiogenic shock, operated on after preoperative stabilization with mechanical circulatory support (VA ECMO or/and IABP or LVAD), the operative mortality was 11%, and overall mortality after one year (including patients with or without surgery) was 33%. The operation was performed at least 10 days after the diagnosis. Complications of ECMO and LVAD in the entire analyzed group included inguinal infections in four patients, thromboembolic phenomena and inguinal infection in one patient, and one device thrombosis.

Ariza-Sole A. et al. reported on 28 patients, 20 of whom qualified for surgical treatment [31]. Fifteen patients were operated on during the first 36 h after admission, four of which required post-operative ECMO support. Five patients were operated on after being stabilized with ECMO at an average 5.2 days after admission. Mortality rate among patients undergoing emergency surgery was approximately 33%, whereas in the second group it was 0%.

The authors highlighted that the patients supported with MCD pre- or postoperatively had a more unfavorable profile according to the INTERMACS scale and a higher risk of adverse events. In addition, ECMO as a bridge to delayed surgery was related to magnificent outcomes in these patients. In two critically ill patients, ECMO was instituted as a bridge to decision; they were eventually not qualified for surgery. Both patients died after several days of support: one of them due to massive respiratory bleeding, the other one due to free-wall rupture and cardiac tamponade.

Ronco D. et al. published the systematic review of 111 studies, including case reports, case series, observational studies, and registries involving 2440 patients [24]. Out of 129 patients stabilized during hospitalization with MCD, almost 100 were on preoperative V-A ECMO. IABP was additionally used in each of the patients, and five of them received Impella. Three patients stabilized with V-A ECMO required additional cannulation of the pulmonary artery or right ventricle due to large shunt and refractory pulmonary edema. In most cases, the operation could be postponed. The period of using MCS was 5.7 days, and mortality rate in patients with pre- and postoperative ECMO instituted was 29.2%. The limitation of this analysis is the lack of precise data for the group, in which ECMO was used preoperatively. Interestingly, in another D. Ronco study [38], preoperative support was related to poorer mortality rate in comparison with emergency surgery, probably because of worse initial hemodynamic status and less frequent qualification for this type of therapy.

Artemiou et al., McLaughlin et al. and Hobbs et al. presented a case series describing three patients each [32,33,34]. Only one patient had a unfavorable result of treatment in the first study [32]. He died after a delayed VSD repair due to a complication associated with implantation of a right ventricular assist device.

In the McLaughlin report, the course of the treatment of one of the patients was unusual: the patient underwent ECMO as a bridge to second surgery due to a failed first VSR repair attempt [33]. The first operation was also delayed due to the response to intensive medical therapy.

Hobbs described the use of VA ECMO and IABP in three patients [34]. The therapy of the first patient, constituting a bridge to transplantation, ended with a hemorrhagic stroke after conversion to a biventricular assist device due to insufficient unloading of the left ventricle. The use of support in the two remaining patients enabled stabilization of patients’ condition and successful surgical treatment.

Sánchez Vega J.D. et al. tested the optimal time for VSR surgery in patients stabilized preoperatively with VA-ECMO (n = 22) [35]. The mortality rate among those operated from day 4 was 36%, whereas it was significantly higher in patients referred for surgery within 1–3 days and 24 h after MCS connection (50% and 62.2%, respectively). The total 30-day death rate among 120 patients admitted in 2008–2018 was 60%, and an attempt of surgical repair was undertaken in 65.8% of patients.

La Torre M.W. et al. were the first to present the treatment results of five patients in cardiogenic shock in the course of acute posterior ventricular septal defect using Impella Recover LP 5.0 Support System as a bridge to surgery [36]. The main duration of this type of support was 14 ± 6 days (we do not know if it includes only preoperative days). The 30-day mortality rate was 40%. The first patient died one day after the surgery because of right ventricular failure. The second one had a massive hemolysis, needed a blood cell transfusion, and died from complications of prolonged intubation. The treatment of the third and fourth patients was successful. The last patient died due to infection and then, during treatment, heavy bleeding from the femoral wound on the 42nd day after surgery.

Gregoric I. et al. presented a case series that included 11 critically ill patients with post-infarcted VSR. Eight patients were implanted with Tandem Heart preoperatively [37]. Each of them survived until the operation, which took place after 7 ± 3 days of support. The 30-day mortality rate was 0%; the six-month rate was 25%. The cause of death was stroke and pneumonia, as well as heart failure leading to multiple organ failure. The mortality rate of the remaining three patients, who qualified for emergency surgery and postoperative circulatory support, was 100%.

Rob D. et al. reviewed 31 patients with VSR. Seven out of fourteen patients with cardiogenic shock received preoperative venoarterial ECMO support due to no improvement with amines and IABP [39]. Mean extracorporeal membrane oxygenation support duration was 12 (±6) days. The early outcome was better than in cardiogenic shock without ECMO group (mortality rate 57.1% vs. 85.71%) The results include the deaths of two patients treated with ECMO who could not wait for surgery due to bleeding. Complications in the ECMO group other than bleeding included infections (seven patients), severe limb ischemia (one patient), and renal replacement therapy (two patients).

In the study of Huang et al., among 47 operated patients, 41 required emergency surgery [40]. Before the operations, 34 were supported with IABP and 6 with VA ECMO. We do not have data as to whether the use of ECMO was associated with delay of surgery. Most diagnosed patients had symptoms typical of Class III and IV Killip–Kimball classification. Urgent operation was related to the mortality at the level of 41%, but among patients with preoperative use of MCS, it was 33%.

A study by M. Matteucci et al. analyzed the pre- and postoperative use of ECMO in 158 patients with mechanical complications of myocardial infarction based on data collected from the Extracorporeal Life Support Organizations’ (ELSO) data registry [41]. From these data, 25 patients out of 67 with various mechanical complications of MI who underwent cardiac surgery had ECMO preoperatively or instituted during surgery. We do not have data on duration of preoperative mechanical circulation; however, the in-hospital mortality rate in groups with ventricular septal rupture, papillary muscle rupture, and free wall rupture treated during surgery or with preoperative ECMO was 60% (15 of 25 patients) (vs. 47.6% with surgery before ECMO support (20/42)). Complications related to pre-and postoperative ECMO (primarily abnormal kidney function and bleeding) occurred in 75.3% of patients.

Fujimoto et al. studied the efficacy of extracorporeal membrane oxygenation in patients with severe circulatory failure due to mechanical complications of MI, resistant to conventional resuscitation [42]. ECMO was instituted in nine patients (four of them suffer from VSR). A favorable course of treatment (surgery, weaned from MCS and discharge) occurred in four patients, including one with VSR.

After analyzing the data on 53 patients with post-infarcted ventricular septal defect complicated by cardiogenic shock, Vondran et al. found that the time from infarction to surgery was the predictor of mortality in the 30-day observation [14]. Performing surgery up to 7 days after the infarction is associated with worse results (OR 5.894; *p* = 0.007); preoperative use of IABP does not reduce mortality rate (of the 36 patients, 16 survived) whereas preoperative use of ECMO tends to improve the early survival rate among patients with post-infarction VSD (used in four patients, three of whom survived).

## 4. Discussion

Early surgery after the diagnosis of the VSR is associated with worse outcomes than the repair performed after 7 days from MI. There are some of possible explanations for this fact. Patients who survive to delayed surgery are in a better preoperative status (critical preoperative state is a known risk factor for cardiac surgery early mortality), even if they were initially in cardiogenic shock. Patients undergoing surgery after 7 days from MI are less likely operated on when in cardiogenic shock either because they are doing well with IABP and catecholamines only, are supported effectively with MCS devices, or are stable for the surgery. Preoperative MCS can definitely help treat cardiogenic shock, which is the other important factor for the mortality of patients with VSR.

The use of MCS as a bridge to delayed surgery allows one to reduce the effect of antiplatelet drugs and have time to reorganize infarcted tissue to achieve better outcomes. Another reason for which the preoperative MCS could be beneficial is the cardiac muscle recovery from MI.

Cardiac surgery in patients with elevated cardiac injury markers due to MI is associated with higher mortality rates. Planned delayed surgery gives more time for diagnostics and is performed electively with higher likelihood of better and more experienced surgeons. The optimal duration of mechanical circulatory support remains unknown. However, researchers seem to be balancing between stabilizing the patient and avoiding serious complications of MCS. In our opinion, the main aim of MCS should be treating shock rather than improving local tissue quality.

This review has several limitations:

- VSR is relatively rare, and it is impossible to conduct a randomized prospective scientific study in this field, therefore the analysis covers a limited number of publications, and the authors emphasize the need for further research.
- Due to the low incidence of mechanical complications of myocardial infarction, limited use of delayed surgery treatment and therapy with MCS, the most important investigated studies include a small number of patients. Analyzed groups cannot be directly compared; nevertheless, they emphasize the presence of certain trends.
- The retrospective nature of research is often related to the limited amount or lack of relevant data.
- Bad outcomes are underreported, which further limits the assessment of the effectiveness of MCS in this difficult patient cohort.
- The methodologies used and the inclusion and exclusion criteria for individual publications often differ significantly; therefore, the results have been summarized and expressed as percentages in order to make approximate comparisons of the obtained results of treatment. The results of the analysis and a summary of collected data can be found in Table 1.

Several publications do not provide data relevant for this review. Some studies reveal incomplete data concerning duration of mechanical circulatory support without precise distinction on pre- and postoperative use of MCS (Mateucci et al. [41], Huang et al. [40], Ronco et al. [38], Rob et al. [39], Vondran et al. [14]) and, in some cases, the temporal possibilities of postponing the surgery. In addition, some retrospective studies include patients not only with VSR but also with other mechanical complications of myocardial infarction (Mateuci et al. [41], Fujimoto et al. [42]). The stage of surgical treatment in individual publications covers a different range of intervention. Due to the limited number of publications on the multistage, delayed surgical treatment of post-infarcted VSR, all these studies have been included.

## 5. Conclusions

The use of mechanical circulatory devices as a bridge to surgery in patients in cardiogenic shock in the course of VSR seems to be a promising direction of treatment. Selecting the most appropriate MCD configuration for unstable patients with myocardial infarction, determining the duration of support and the most appropriate timing for surgery in patients with VSD requires further research.

## Figures and Tables

**Table 1:** Publication summary.

Study	Type of Treatment	Number of Patients Qualified for the Procedure	VSR Diagnosis to Surgery	MCS Duration before Surgery	Early Mortality		
					n	%	
1. Morimura H. et al., 2020 [17]	Delayed surgery with preoperative ECMO and IABP	8	1.9 days	36.9 h (ECMO) 43.2 h (IABP)	1		12.5%
					3 during 2 years		37% during 2 years
2. Malik J. et al., 2021 [30]	Delayed surgery with preoperative ECMO or/and IABP or LVAD	27	18.8 days	13.2 days	3		11% (operative mortality)
					9 (overall mortality after one year from any cause)		33% (overall mortality after one year from any cause)
3. Ariza-Sole A. et al., 2020 [31]	Delayed surgery with preoperative ECMO	5	5.2 days	~5 days	0		0%
		5 + 2 (+ECMO as a bridge to decision)			2		28.5% (mortality including patients on ECMO as a bridge to decision)
	Urgent surgery without/with postoperative ECMO	15			5		33%
4. Ronco D. et al., 2021 [24]	Delayed surgery with preoperative ECMO or ECMO in combinations with other MCS (+Impella; +PA/RV cannula; +IABP)	100	~ 6.3 days	5.7 days (ECMO)			29.2%
5. Artemiou P. et al., 2020 [32]	Delayed surgery with preoperative ECMO	3	1st patient: 13 days	1st patient: 12 days	1 (third patient)		33.33%
			2nd patient: 17 days	2nd patient: 17 days			
			3rd patient: 11 days	3rd patient: 9 days			
6. McLaughlin A. et al., 2016 [33]	Delayed surgery with preoperative ECMO	3 1 patient with VSR+PMR	1st patient: 4 days	1st patient: no data	0		0%
			2nd patient: no data	2nd patient: 7 days			
			3rd patient: 9 days	3rd patient: 5 days			
7. Hobbs R et.al., 2015 [34]	Delayed surgery with preoperative ECMO (+IABP)/BIVAD	3	1st patient: 2 days	2 days	1 (after conversion to BIVAD)		33.3%
			2nd patient: 11 days	7 days			
			3rd patient: 5 days	4 days			
8. Sanchez Vega J.D. et al., 2020 [35]	Surgery performed from 4th day with preoperative use of ECMO	No data	an average of 5 days [1,2,3,4,5,6] in all 3 groups with ECMO	4 days			36%
	Surgery performed within 1–3 day with preoperative ECMO	No data		1–3 days			50%
	Surgery performed within 24 h with preoperative ECMO	No data		within 24 h			62.2%
	All types of treatment	122	2.6 ± 3.5 days				60%
9. La Torre et al., 2011 [36]	Delayed surgery with preoperative Impella Recover LP 5.0	5	No data	14 ± 6 days	2		40%
10. Gregoric ID et al., 2014 [37]	Delayed surgery with preoperative Tandem Heart	8	No data	7 ± 3 days	0		0% within 30 days
11. Ronco D. et al., 2021 [38]	Surgery with preoperative ECMO	35	No data	No data	19		54.28%
	Urgent surgery	212	No data	No data	108		50.94%
12. Rob D. et al., 2017 [39]	Delayed surgery with preoperative ECMO	7	No data	Mean duration of ECMO support was 12 (±6) days (no data if it includes only preoperative period)	4		57.1%
	Patient in cardiogenic shock treated without preoperative ECMO	7			6		85.71%
13. Huang S.M. et al., 2015 [40]	Surgery with preoperative ECMO	6	No data	No data	2		33%
	Urgent surgery	41			17		41.46%
	All the patients with VSR	47	No data (AMI to VSR repair 5.3 ± 10.4 days)		17		36.2%
14. Matteucci M. et al., 2020 [41]	Surgery with pre- or intraoperative ECMO (VSR+other AMI mechanical complications)	25	No data	No data	15		60%
	Surgery with postoperative use of ECMO (VSR+other AMI mechanical complications)	42	No data	No data	20		47.6%
	VSR group with pre- and postoperative ECMO with and without surgery	102	No data	208.2 ± 242.5 h	66		64.70%
	All the patients with AMI mechanical complications with or without surgery with pre- or postoperative MCS	158	No data	5.9 days	99		62.70%
15. Fujimoto K. et al., 2001 [42]	Surgery with preoperative ECMO in patients with a critical general condition (VSR+ other AMI mechanical complication)	9		76 ± 5.7 h	5		55%
16. Vondran M. et al., 2021 [14]	Surgery with preoperative ECMO	4	No data	No data	1		25%
	Surgery with preoperative IABP	36	No data	No data	20		55.55%
	Urgent surgery	32			23		71.87%
	All patients with VSR	53	No data (AMI to VSR repair 11.9 ± 10.6 days)		23		56.6%

## Data Availability

Not applicable.
